# Applying adaptive management and lessons learned from national assessments to address logistical challenges in the National Wetland Condition Assessment

**DOI:** 10.1007/s10661-019-7320-8

**Published:** 2019-06-20

**Authors:** D. J. McCauley, W. J. Arnold, J. B. Saxton, C. J. Turner

**Affiliations:** Great Lakes Environmental Center, Inc., Traverse City, MI USA

**Keywords:** Adaptive management, Logistics, Field logistics, National environmental assessments, National Aquatic Resource Surveys, NARS, National Wetland Condition Assessment, NWCA

## Abstract

The National Wetland Condition Assessment (NWCA) is one of a series of probability-based National Aquatic Resource Surveys (NARS) conducted by the U.S. Environmental Protection Agency (USEPA) to provide a comprehensive assessment of the condition of the Nation’s waters. Randomized design and standardized training and protocols allow USEPA to analyze data that are nationally consistent and regionally relevant. Each NARS assessment was preceded by careful consideration of key logistical elements that included pre-survey planning, training, sampling logistics, and laboratory analysis. Numerous state, tribal, and contractor crews were supported across the country for each assessment; sampling and sample analyses were tracked from initiation; laboratory analyses were completed at USEPA, state, regional, and contract laboratories; and the data analyses and reporting were completed by USEPA-led workgroups, states, and contractors. The complexity and difficulty of each step offered unique challenges and provided lessons learned for each of the NARS assessments. Major logistical elements for implementing large scale assessments that are constrained by sampling period and number and duration of visits are covered in this paper. These elements include sample transport, equipment and supplies, sampling and sample tracking, information management regional technical expertise, and a sound field training program. This paper describes how lessons from previous assessments were applied to the NWCA and how new challenges faced in the NWCA were addressed and carried forward into future surveys.

## Introduction

**Fig. 1:**
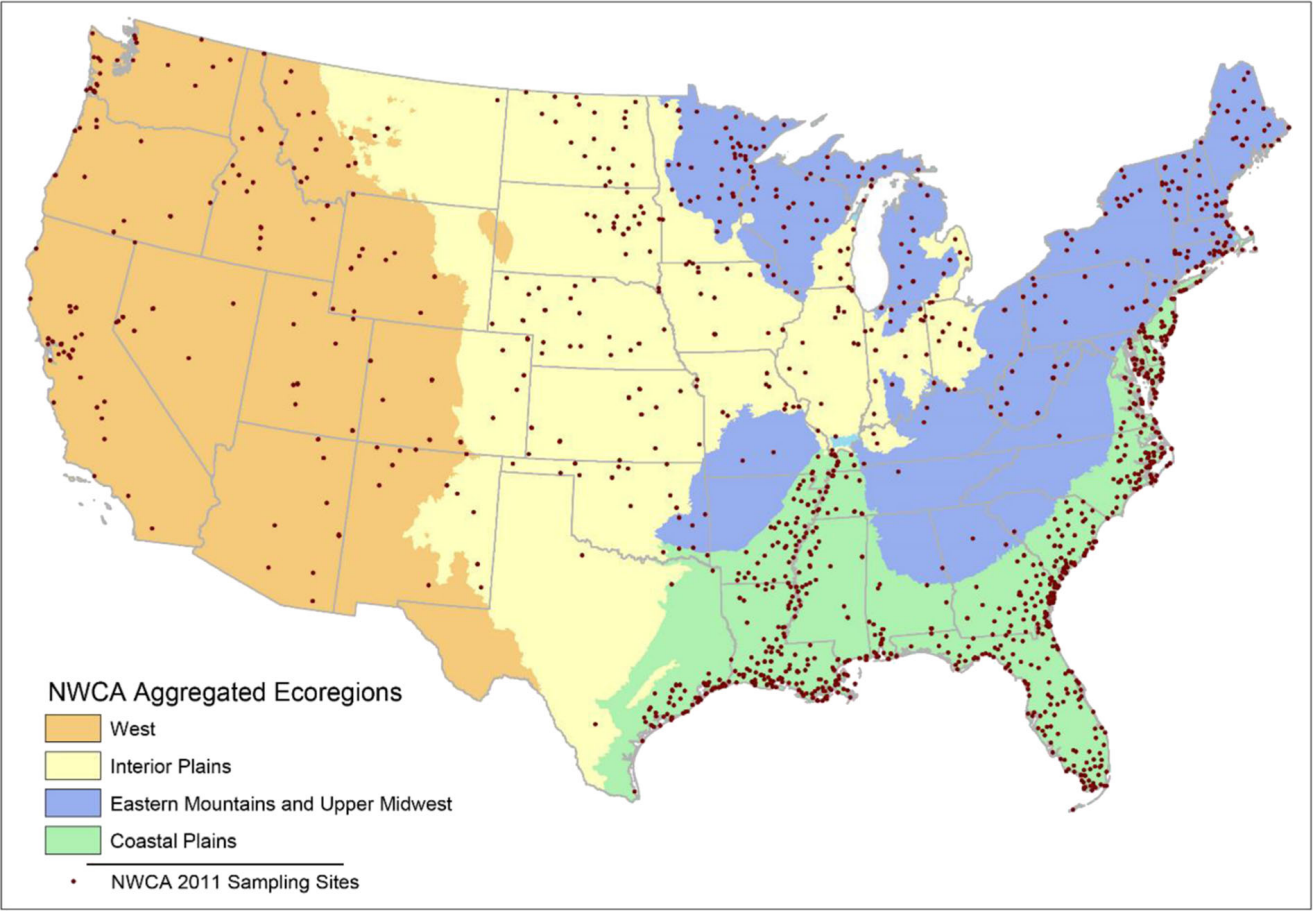
Distribution of the 1179 NWCA 2011 sampling sites and NWCA aggregated ecoregions (adapted from USEPA 2016)

The 2011 National Wetland Condition Assessment (NWCA) was the first-ever nationwide probabilistic survey of U.S. wetlands. The NWCA is one of four national assessments under the U.S. Environmental Protection Agency’s (USEPA) National Aquatic Resource Surveys (NARS), which are designed to generate statistically valid estimates of the ecological health of national water resources through sampling for representative biotic community, water chemistry, and associated ecological data. The NWCA rotates on a 5-year schedule with the other NARS assessments: the National Lakes Assessment (NLA), National Rivers and Streams Assessment (NRSA), and the National Coastal Condition Assessment (NCCA) (Table 1). The need for nationwide surveys that could generate data on aquatic resource conditions at national and regional scales was identified by several entities, beginning with Paulsen et al. (1998), Shapiro et al. (2008), GAO (2000), NRC (2000), and the H. John Heinz Center for Science, Economics and the Environment (2002). The 2004 Wadeable Streams Assessment (WSA) was a national-scale precursor to the NARS program that developed many of the methods and approaches used in the NARS assessments (Table 2). Program support contractors for the WSA and NARS assessments were tasked with coordinating and supporting multiple states, agencies, tribes, and contractors for the design and execution of survey training and logistics. Each survey has typically involved between 50 and 80 field crews and some 1200 sampling locations that are surveyed following standardized protocols (Table 3). The NWCA 2011 fielded 57 field crews from 49 organizations and sampled 1179 sites with 1275 field visits (Fig. 1 and Table 3). In preparing for implementation of the NWCA 2011, USEPA and the supporting contractors had the opportunity to apply a number of lessons learned from previous national assessment field operations. As with all of the assessments, the complexities and “on the ground” realities of the NWCA execution also presented logistical challenges that required implementation of adaptations from previous surveys and adaptive management during the field season to solve problems not anticipated in planning phases.

**Table 1:**
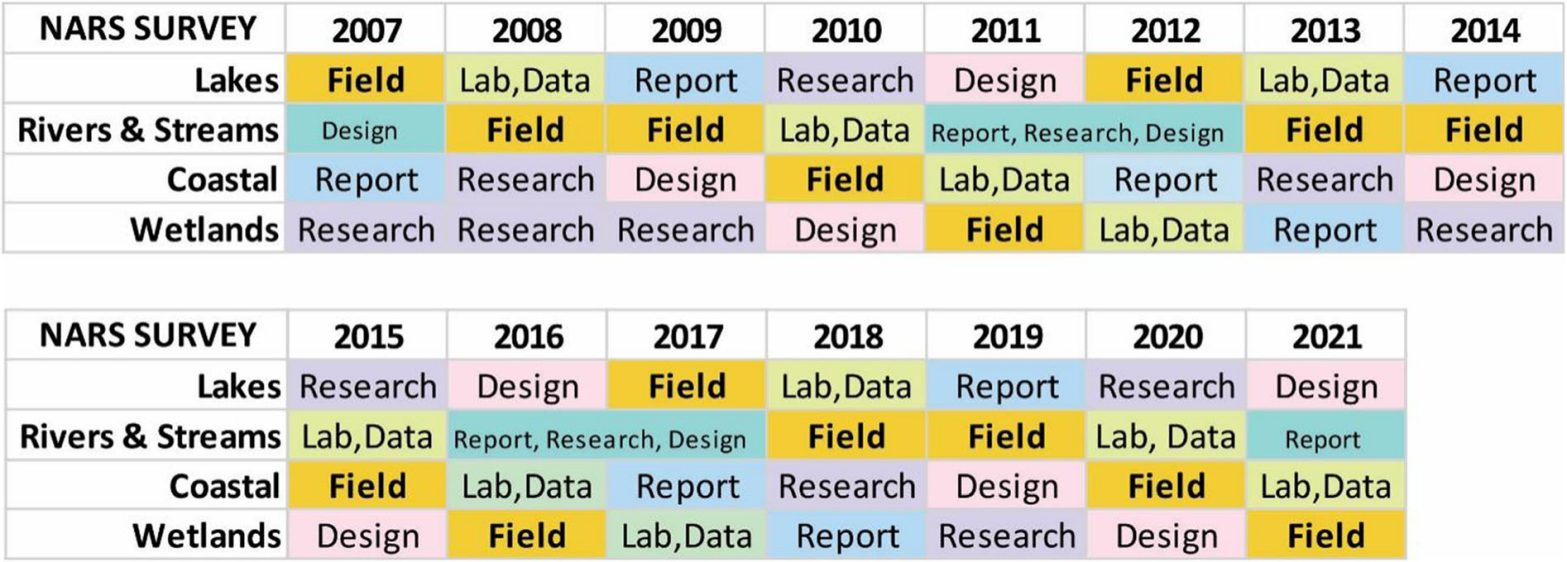
USEPA National Aquatic Resource Surveys (NARS) program schedule by year

**Table 2:** USEPA national assessment field seasons since 2004

Field season	National assessment
2004	Wadeable Streams Assessment
2007	National Lakes Assessment
2008–2009	National Rivers and Streams Assessment
2010	National Coastal Condition Assessment
2011	National Wetland Condition Assessment
2012	National Lakes Assessment
2013–2014	National Rivers and Streams Assessment
2015	National Coastal Condition Assessment
2016	National Wetland Condition Assessment
2017	National Lakes Assessment
2018–2019	National Rivers and Streams Assessment
2020	National Coastal Condition Assessment
2021	National Wetland Condition Assessment

**Table 3:** NARS field season statistics and sampling periods since 2011

	Number of field crews	Number of organizations	Number of sites	Number of sampling events	Sampling period
National Wetland Condition Assessment (2011)	57	49	1179	1275	April 4–Nov 2, 2011 (8 months)
National Lakes Assessment (2012)	89	51	1139	1235	May 2–Sept 28, 2012 (5 months)
National River and Streams Assessment (2013)	63	44	966	1054	April 30–Oct 2, 2013 (5 months)
National River and Streams Assessment (2014)	77	46	1175	1283	April 28–Nov 19, 2014 (7 months)
National Coastal Condition Assessment (2015)	53	27	1438	1549	June 1–Oct 27, 2015 (5 months)

## Logistics in national assessments

Hughes and Peck (2008) defined logistics as the tactics by which the gear, samples, and data for the selected indicators are moved between the supply sources, central laboratories, and the field. Logistics were further defined by Hughes and Peck (2008) as one of three secondary constraints affecting extensive aquatic resource surveys, the others being survey design and indicators. Primary constraints are management objectives, schedules, and funding. According to Hughes and Peck, logistical constraints are:

- Sampling period (index period);
- Sampling visits—number and duration;
- Sample transport;
- Equipment and supplies;
- Information management;
- Technical expertise of crews; and
- Field training.

We also identify “Site Evaluation and Replacement” as a constraining element of logistics in addition to those outlined by Hughes and Peck. Site evaluation is the process of determining if a site meets the definition of the given survey’s target population and then the qualification of target sites as being both physically viable (i.e., meeting the site evaluation guideline criteria) and accessible. Site evaluation is accomplished through both pre-survey desktop evaluations and actual field visits. Site evaluation also includes the replacement of non-target or un-sampleable sites with replacement sites following a rigidly defined replacement plan. Because the probabilistic survey design is an automated GIS (Geographic Information System) process using digital maps and imagery, real-time conditions can vary from digital representations. For example, a lake that appears on a topographic map or satellite image may actually be dry during the sampling period or a wetland area digitized in the 1980s may have come under agricultural use in subsequent years. For example, Stoddard et al. (2005a) reported that 207,770 km, or 33% of the mapped perennial stream length in USEPA’s River Reach File in the western USA, was non-perennial or not a true stream and, therefore, not part of the target population for NRSA. The level of error varied from one climatic region to another: 55% in the xeric region, 33% in the plains, and 24% in the mountains. While each survey has experienced different levels of removal and replacement of initial target sites after site evaluation, the outcomes have always proven to be a significant constraint. Replacing target sites can have a compounding effect on logistics, such as repeating site evaluation and access permission activities, diverting field crews from planned travel routes, and completing sampling within the defined sampling time frame.

## The importance of adaptive management

Contractors and collaborating organizations have applied adaptive management under USEPA guidance to address logistical challenges encountered during survey sampling periods. Simply defined, adaptive management is decision making to deal with uncertainty or unanticipated events while monitoring a system, through (a) testing assumptions, (b) adaptation, and (c) learning. Adaptive management has been critical to address the real-time logistical challenges of the national assessments while keeping within index period schedules and program budgets. Most importantly, the lessons learned and solutions applied during the surveys must be considered and applied to subsequent surveys. In the following narrative, the authors summarize examples of logistical challenges and adaptive management leading up to the NWCA 2011, discuss specific NWCA 2011 challenges and outcomes, and then outline adaptations from the NWCA 2011 and other NARS surveys leading up to the NCCA 2015.

USEPA’s WSA and NARS national assessment programs evolved from the Environmental Monitoring and Assessment (EMAP) administered by USEPA’s Office of Research and Development (Stoddard et al. 2005a, b). EMAP developed monitoring and assessment tools, established approaches for reporting on ecological status and trends, and cultivated inter-agency cooperation on national- and regional-level assessment projects. EMAP collected field data from 1990 to 2006 while the WSA survey was conducted in 2004. Many of the tools, methods, and organizational approaches from EMAP were used in the 2004 WSA assessment.

A principal development in the NARS surveys since the EMAP and WSA surveys has been the expanding role of contractor organizations to facilitate training and logistics for each field season, in addition to more traditional functions like field sampling and laboratory analyses. Beginning with the NLA 2007, leading up to the NWCA 2011, and continuing to present day, contractor support has grown to include the following logistical functions:

- Contractor Field Logistics Coordinator and base support
- Field demonstration studies
- Adaptive training
- Site evaluation and replacement
- Information management, communications, and tracking

In the following sections, the authors discuss how growth within these functions through lessons learned and adaptations has controlled and reduced logistical constraints presented by the NWCA and surrounding national assessments.

### Contractor Field Logistics Coordinator and base support

The Contractor Field Logistics Coordinator (CFLC) and base support functions began with the 2007 NLA as part of the official program organization. These functions are provided by a logistics contractor and have evolved and expanded with each subsequent NARS survey. As part of providing an expert individual to fulfill the CFLC role and provide base support, the logistics contractor assists USEPA with the following critical tasks before, during, and after each field season:

- Developing supply and equipment lists for base kits (re-usable supplies and equipment) and site kits (consumable sampling supplies);
- Selecting supply and equipment vendors that provide best value along with correct and timely shipments;
- Ensuring that supply and equipment orders meet index period sampling schedules;
- Overseeing packaging plans for base kits and site kits;
- Developing checklists to ensure the accuracy and completeness of base kits and site kits;
- Overseeing the delivery of equipment and supplies to field crews before and during the field season, including the fulfillment of both planned and urgent requests;
- Tracking supply inventories;
- Developing shipping plans for the delivery of samples from crews to analytical and batch laboratories and from batch laboratories to analytical laboratories;
- Tracking the status of field activities on a daily basis (e.g., sites sampled/dropped, samples collected, missing samples);
- Maintaining communication with all field crews to ensure consistent field crew operations; and
- Overseeing sample shipments from field crews.

The role of the individual CFLC is to assist USEPA in coordinating the following critical tasks before, during, and after the field season by:

- Continually monitoring the CFLC’s telephone and email contacts, including evenings and weekends. This ensures that field crews obtain timely assistance, including during on-site operations. This service minimizes downtime and reduces field-related errors;
- Designating an alternate CFLC if he/she is temporarily unavailable (e.g., due to vacation or illness). Set up automated responses to the phone line and email to alert crews to contact the alternate CFLC;
- Responding to calls or emails from crew members as soon as was practical. During weekday business hours, response time is typically less than 2 to 3 h. Every effort is made to respond immediately if the crew is at the sampling site;
- Referencing Field Operations Manual (FOM) (USEPA 2011a, b) and training materials when answering questions to ensure accurate and consistent responses. Referring questions to the appropriate USEPA staff member or trainer if answers are not specified in program documents or if unsure of the answer. Referring questions to the appropriate national or state laboratory, USEPA Information Management (IM) staff, or Regional Monitoring Coordinator, and copying USEPA Program staff on email traffic when appropriate;
- Leading a regularly scheduled (typically biweekly) conference call involving all field crews to provide a forum to ask questions, learn about other crews’ experiences, and receive guidance updates; and
- Alerting appropriate USEPA staff members immediately by email and/or phone if major issues arise during field operations and relaying USEPA’s corrective action to the crew as soon as possible. Providing a summary to USEPA of any incidences and their resolutions.

The logistics contractor and CFLC apply adaptive management to help develop improvements to operations and methods for each survey field season. Improvements and adaptations are conveyed to field crews through the Field Operations Manual, Quick Reference Guide, field training, and, ultimately, implementation in the field. The logistics contractor and CFLC are also prepared to help USEPA evaluate field methods for survey-specific parameters and develop field methodologies through demonstration studies (discussed in a following section of this document). In the following paragraphs, specific examples of these lessons learned and adaptations pertinent to NARS and the NWCA are provided.

As national assessments were implemented, USEPA and its contractors began to recognize that having a routine platform for answering questions, reinforcing protocols when problems were detected, and allowing crews to share insights gained from practical experience proved to be an invaluable aid to efficient operations. As an adaptation to this lesson learned, regular field crew calls involving all field crews have become standard for NARS surveys. The addition of field crew calls throughout the summer of the NWCA 2011 facilitated answering questions and alerting crews to common mistakes in shipping or application of protocols. Given the fact that there were more than 50 crews sampling as part of the NWCA 2011 survey, these calls provided a much-needed and convenient forum for crews to seek information and to ensure that all crews were receiving uniform instructions.

The development of contractor-provided base support was another useful lesson learned and adaptation. During the initial NARS surveys, USEPA staff were responsible for base support: the coordination of all requests and deliveries for crew supplies and equipment. In preparing for the NWCA 2011, USEPA and the logistics contractor used information from past surveys to conclude it would be more effective to have the logistics contractor perform the base support function. Due to its operational flexibility and constant availability, the logistics contractor was better able to respond to urgent field crew requests, resulting in fewer sampling schedule delays and improvements in overall logistical efficiency.

The establishment of a “batch laboratory” hub operated by the logistics contractor was another principal lesson learned and adaptation. During the early national assessments, all samples were shipped directly to each analytical laboratory from the crews. Because of the number of samples and laboratories involved with the assessments, shipping samples was time consuming and often confusing for the crews and led to shipping errors. Learning from this lesson, the NWCA 2011 began a process of having crews ship some samples to a central batch laboratory, which in turn shipped the samples to the correct analytical laboratory. This adaptation was adapted in future NARS assessments and has resulted in fewer misplaced samples.

The logistics contractor has applied adaptive management techniques to modify existing equipment or have items custom built to best meet program objectives. In 2011, the logistics contractor supported the NWCA by furnishing supplies to 57 crews that completed 1275 sampling events over 7 months. This survey required a base kit that contained a number of specialty items including a plant press, Pulaski axe, laser rangefinder, compass, and framed backpack. The logistics contractor sought out items that would function well and last in the field while costing the government the least amount possible. The NWCA 2011 survey also required a number of items that were not commercially available and items that needed to be adapted to the sampling protocol:

- For the collection of the sediment enzyme and soil isotope samples, 60-cc plastic syringes were modified for the collection of surficial sediment.
- To ensure the safe shipment of plant voucher specimens to a national laboratory, the logistics contractor hired a container manufacturer to custom build plant folders and shipping boxes.
- The logistics contractor designed an improved bulk density sampler and then engaged a steel fabrication company to custom manufacture the new stainless steel samplers. Contractor field researchers had determined that the bulk density sampler specified in the first version of the NWCA 2011 FOM (a tin can) would not have been durable enough to last for a field season and variations in tin can sizes would yield varying sample volumes. The new sampler was successfully employed for the 2011 survey season and will be used again for subsequent NWCA surveys.

### Field demonstration studies

As previously mentioned, the logistics contractor has supported the evaluation and improvement of field methods through field demonstration studies. NWCA 2011 logistics were optimized prior to the field season through an on-site field demonstration and/or proof of concept study that served to vet the protocol, finalize field materials and equipment, and act as a demonstration that the protocol could be completed within an acceptable time frame (e.g., 8–10 h field day). Hughes and Peck (2008) identified “field trials” (i.e., field demonstration studies) as important design elements that are conducted by contractor training facilitators and national experts. The NWCA field demonstration served to refine and finalize the field protocols, field forms and labels, and other survey elements. The NWCA protocols and many of the indicators were introduced at a national wetland workshop, followed by an “in the field” proof of concept assessment, and further refined during a “Train the Trainer” workshop, designed to train multiple trainers prior to the regular training season on protocols specific to the NWCA assessment. These pre-survey planning exercises served to make the logistical implementation of the upcoming survey field season successful in terms of having an established protocol, capable trainers and facilitators, and a realistic workflow plan for the field work. Because of the extensive improvements adapted from the NWCA 2011 demonstration studies, subsequent NWCA field demonstrations will likely be less extensive and serve to develop more routine improvements such as updating field forms and developing new indicator protocols. The lessons learned and adaptations from the NWCA 2011 field demonstrations will continue to benefit future NWCA survey implementation.

### Adaptive training

Field crew training is essential to the successful implementation of a “large-scale” national assessment such as the NWCA. Since 2007 and including the NWCA 2011, the coordination and facilitation of the NARS training has been the responsibility of the logistics contractor. Like all of the national assessments, the NWCA requires the use of standardized protocols so that field crews collect samples and site data exactly the same way. Because consistency in how a protocol is taught is absolutely necessary, protocols must be written so that they are applicable in each ecoregion. The concept sounds simple enough; however, conveying this consistency through training is confounded by varying on-site conditions found in different US ecoregions (Fig. 1), varying crew experience levels, and each trainer’s understanding of specific protocols. Training consistency requires that each trainer be sufficiently versed in the protocol to be able to apply it to various ecoregions and environments. They must also have familiarity with regional characteristics likely encountered and be able to respond to crew questions.

Ideally, the same set of trainers could train at all training events and simultaneously have expert knowledge of conditions in each ecoregion. Since this is not practical, some additional regional expertise is needed to effectively train on soils and plant protocols. To address this training need, regional experts are used even though this may affect consistency by introducing regional protocols. For example, in the NWCA, there are four principal training areas: water quality/buffer zones, soils, physical habitat, and wetland plant identification. Each training area also has a significant post-sampling activity that includes sample preparation and shipment of the samples or data to a state or national laboratory. In the NWCA, water quality/buffer zone and physical habitat data collection and post-sampling protocols are relatively similar across all ecoregions, whereas soils and wetland plant identification protocols are very ecoregion specific and require the assistance of regional experts. This issue has been addressed through the adaptation of using regional NRCS soil experts and local expert botanists for the trainings. A lesson learned, however, is that regional experts often tend to teach protocols with which they are most familiar, may not be familiar with standard NARS protocols, and may even inject their own preferences into the training. To adapt to this need for both nationally consistent and regionally specific training, USEPA and the logistics contractor incorporated a new training role as part of NWCA 2011: the Training Facilitator. The role of the Training Facilitator is to oversee each training module and field practice and offer guidance to the specific trainer regarding the intent of the protocols and sampling methods. The role of Training Facilitator is not to serve as an expert in each indicator, but as an expert in all protocols to ensure that they are taught consistently in all ecoregions and site-specific situations. The Training Facilitator is also available to follow up on questions or to contact USEPA directly with site-specific questions on how protocols should be interpreted.

### Site evaluation and replacement

As explained in previous sections, we identify site evaluation and replacement as both a logistical constraint and an important logistical function that has benefited from lessons learned and adaptive management. The effectiveness of site evaluation tools and methods are critical to meeting Agency goals, and the incidence and timing of site removal and replacement has a direct effect on maintaining survey schedules within the index period. Sites must be qualified as both physically sampleable, meeting criteria like depth, area, or composition, and accessible, both physically and legally. Ideally, sites are qualified in the pre-visit desktop site evaluation and landowner identification and permission processes before crew deployments. Removing sites after scheduled field visits results in lost effort and possible disruption to the sampling schedule.

One example of an important adaptation from the predecessor WSA program was applied to the NWCA and subsequent NARS surveys: a more defined and repeatable site evaluation process for identifying property owners and obtaining access permission. Sites often require permission from landowners to either traverse the owner’s property to reach a site or to sample a site directly on the property. Besides basic permission, some owners require documentation like special permits or liability waivers before granting access so identifying landowners prior to or the field season and/or scheduled visit is essential to efficient logistics. To facilitate identification of property owners, WSA contractors developed a detailed decision flowchart for identifying and qualifying property owners. The flowchart detailed the most efficient steps for identifying parcels, communicating with local government entities, and finding contact information for parcel owners. By defining the process in a straightforward flowchart, contractors were able to assign the property identification and permission task to multiple technicians with minimal training and oversight, thus speeding the site qualification process and helping the survey stay within the sampling schedule. This process is especially important for wetland sites, since they are often on private land or surrounded by private parcels that require permission to cross. The landowner site evaluation flowchart and process developed in the WSA was adapted to the NWCA 2011 and was essential to improving an often complicated and drawn-out process. And, to effectively communicate with landowners in the NWCA 2011 (particularly landowners concerned with the implications of a government assessment), the logistics contractor found adapting the general script that USEPA provided in the site evaluation guidelines (USEPA 2011b) to be very helpful. Contractor field crews were also able to support pre-visit landowner site evaluation support by contacting local jurisdictions and landowners.

Another important component of site evaluation and replacement is the need to obtain scientific collectors permits from state, tribal, and federal agencies. These permits usually require reporting information like site lists, sampling locations, and expected species months in advance of the planned sampling date. Consequently, the site evaluation steps described above need to be completed prior to the application for scientific collectors permits.

Geospatial map and imagery reference data compiled in a GIS are also important site evaluation resources. The automated process that USEPA uses to develop the target population utilizes standard GIS data such as the National Hydrography Dataset. As the NARS surveys have progressed, the logistics contractor has learned to adopt rigorous GIS desktop evaluations of individual sites utilizing the latest reference layers and map services currently available to analyze underlying data such as land cover/land use, hydrography, and ownership. The rapid improvements in the accuracy and resolution of publically available GIS data facilitate real improvements in desktop site evaluation effectiveness as each survey season repeats. Advancements in geospatial data delivery have consistently improved the speed and efficiency of the desktop site evaluation process through all of the NARS assessments including the NWCA.

In the NWCA 2011, many sites were dropped not as a result of desktop reconnaissance but after the scheduled on-site visit. Because of the extra time and resources and schedule disruptions caused by visiting sites only to drop them, this proved a major constraint on logistics, program efficiency, and budgets and threatened schedule compliance for the summer index period. Compared with other waterbody types, wetlands have some unique characteristics affecting whether the site is sampleable and accessible. A lesson learned during NWCA 2011 was that the more pre-visit site evaluation tools and landowner communications are effectively adapted and applied, the more likely crews will be able to perform within the index period sampling schedule.

### Information management, communications, and tracking

Information management is the most pervasive logistical constraint in national assessments. The successful management of all logistical constraints and functions is dependent on effective communication tools and practices. Growth in the logistical functions of information management, communications and tracking through lessons learned and adaptations have greatly improved NARS survey efficiencies since their inception. Successful communication among the many organizations involved in national assessments (i.e., federal and state agencies, contractors, laboratories, suppliers, etc.) is vital to assessment success. As an example of the scope of information management needed for national assessments, the number of organizations just providing field crews for NARS surveys has ranged as high as 51 (Table 3).

The logistics contractor’s role supporting information management has increased through each NARS assessment and includes working daily with field crews, the public, and USEPA’s Information Management (IM) Team to facilitate survey logistics during field seasons. The lines of communication must be established well before the field season. As stated earlier, communication with field crews continues throughout the field season and the CFLC serves as the main point-of-contact for fielding crew questions and resolving problems.

Important adaptations in logistics information management have been made through improved shipping and tracking methods. In one example, developed during the NCCA 2015, sample labels, tracking forms, and pre-printed FedEx® shipping labels for each site and sample event are identified with a shipping group number (e.g., T1, T2, and T3). Field crews simply need to match the T# on the sample labels, tracking forms, and shipping labels to assure that the correct samples and paperwork are shipped to the appropriate laboratory. The pre-paid shipping labels also identify the sample types and preservation type (e.g., wet ice, dry ice). Spreadsheets are used to track quantities for site kit materials and were updated throughout the season to track the quantity of items on order and in inventory. This adaptation had the immediate effect of reducing sample shipping errors compared with previous surveys and will benefit the 2016 NWCA and future NARS survey logistics.

**Fig. 2:**
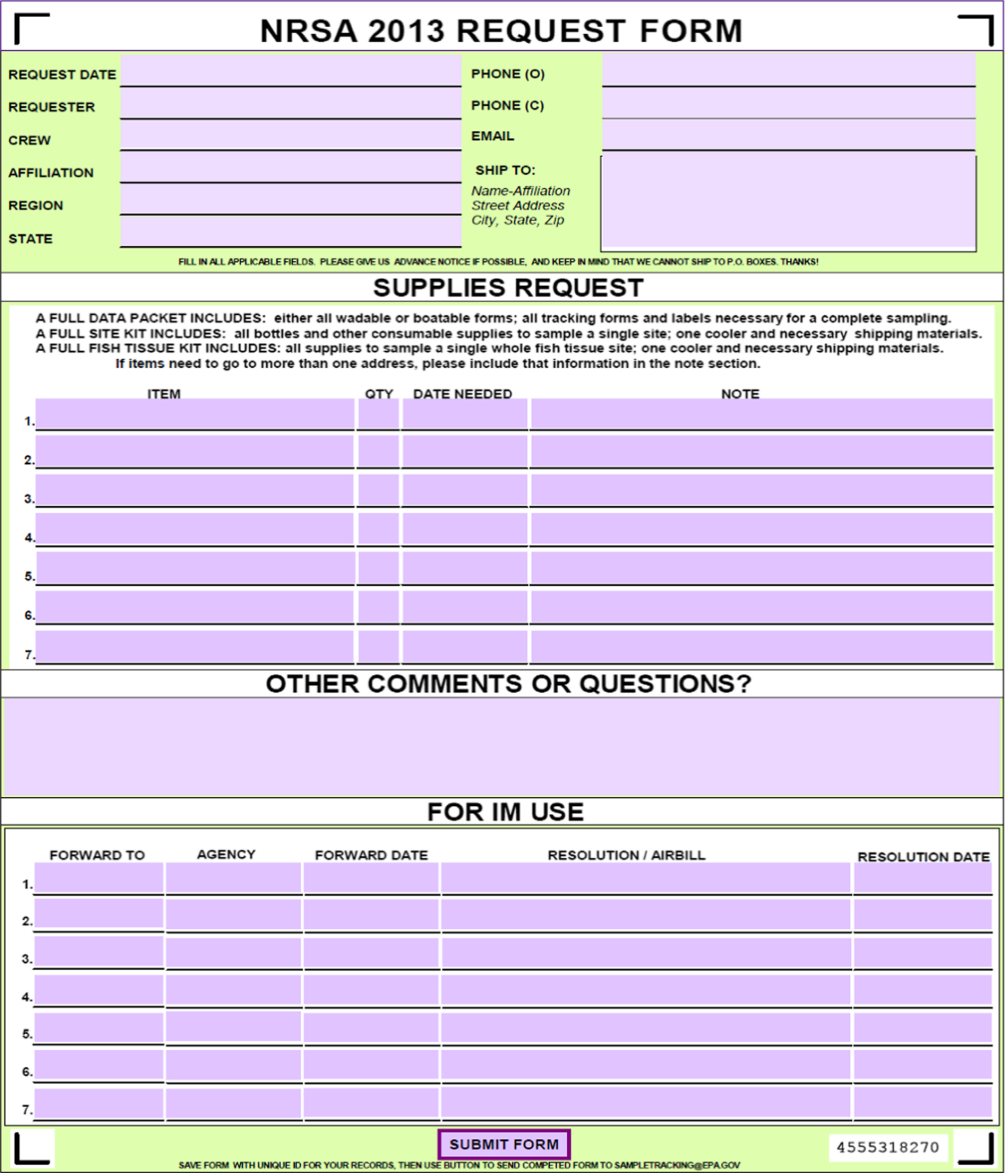
Fillable PDF supply request form

Another information management improvement was the development during the NWCA 2011 of a fillable PDF form for crews to use when ordering supplies (Fig. 2). This request form contains a drop-down menu of commonly requested items, as well as an area for crews to type any other item they need. The request form is emailed by the field crew to a central email address where the USEPA IM Team receives it and enters the requested items and other data into the IM Database. A series of spreadsheet updates from the IM Database allow the logistics contractor to easily determine which orders had been fulfilled and which items still needed to be shipped. While the fillable PDF form provides accuracy, convenience, and control, there are instances when its use may not be practical. During a survey season, the logistics team has learned that crews may encounter an urgent need for supplies when an item is lost or broken, when supplies run out unexpectedly, or when sampling schedules abruptly change. In these instances, logistics procedures have adapted to allow crews bypass the request form and communicate the request directly to the logistics contractor CFLC who then immediately fills and tracks the order.

The logistics contractor has collaborated with shipping companies to improve survey logistics through new adaptations to shipping labels and tracking systems. For example, during the NWCA 2011, the logistics contractor developed an adhesive pre-printed label design that no longer required the field crew to fill-in and complete the label. There was, however, an additional label that was needed for Saturday delivery, and the method by which the labels were printed in bulk (e.g., 1 of 50 labels) often caused confusion on the part of the FedEx employee printing the labels, which could, in turn, delay field crew progress. As an adaptation to address this lesson learned, in 2014, the logistics contractor worked with FedEx to introduce a bulk “return” label. This label was easily processed by FedEx employees, required no special steps on the part of the field crew, and was automatically flagged for Saturday delivery when needed.

With the change to bulk return labels in 2014, the CFLC also instituted automated email notices from FedEx every time a package was shipped, delivered, or encountered a delay. This immediate feedback allowed the CFLC to contact FedEx and/or the field crew to quickly rectify problems and improve on-time sample delivery. In the event of a delay, the CFLC would initiate a “trace” through FedEx and upgrade the shipment to Saturday delivery if needed. With the adaptation of this new protocol, the CFLC can intervene in cases where a shipment is delayed and avoid delays that previously could carry over into the following week.

**Fig. 3:**
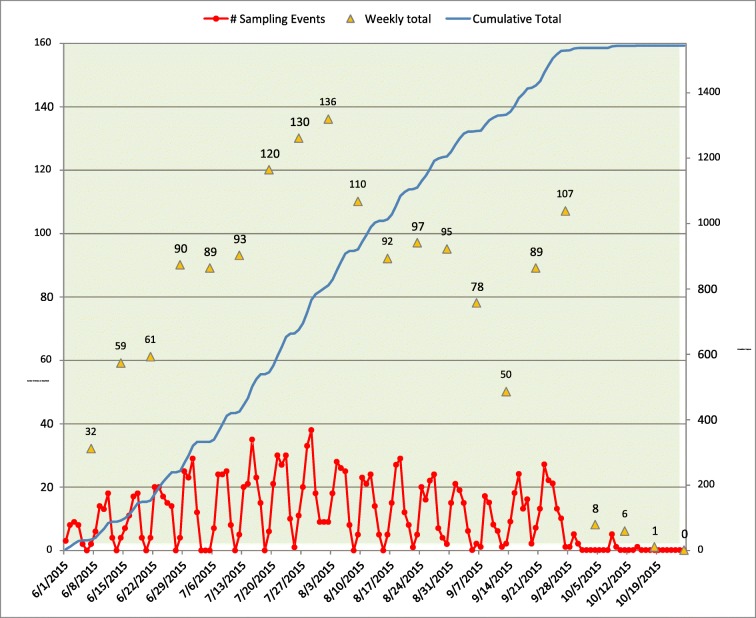
NARS sampling events per day/week/season

**Fig. 4:**
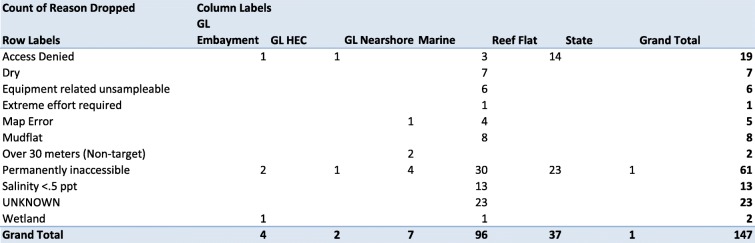
Screenshot of dropped sites summary

**Fig. 5:**
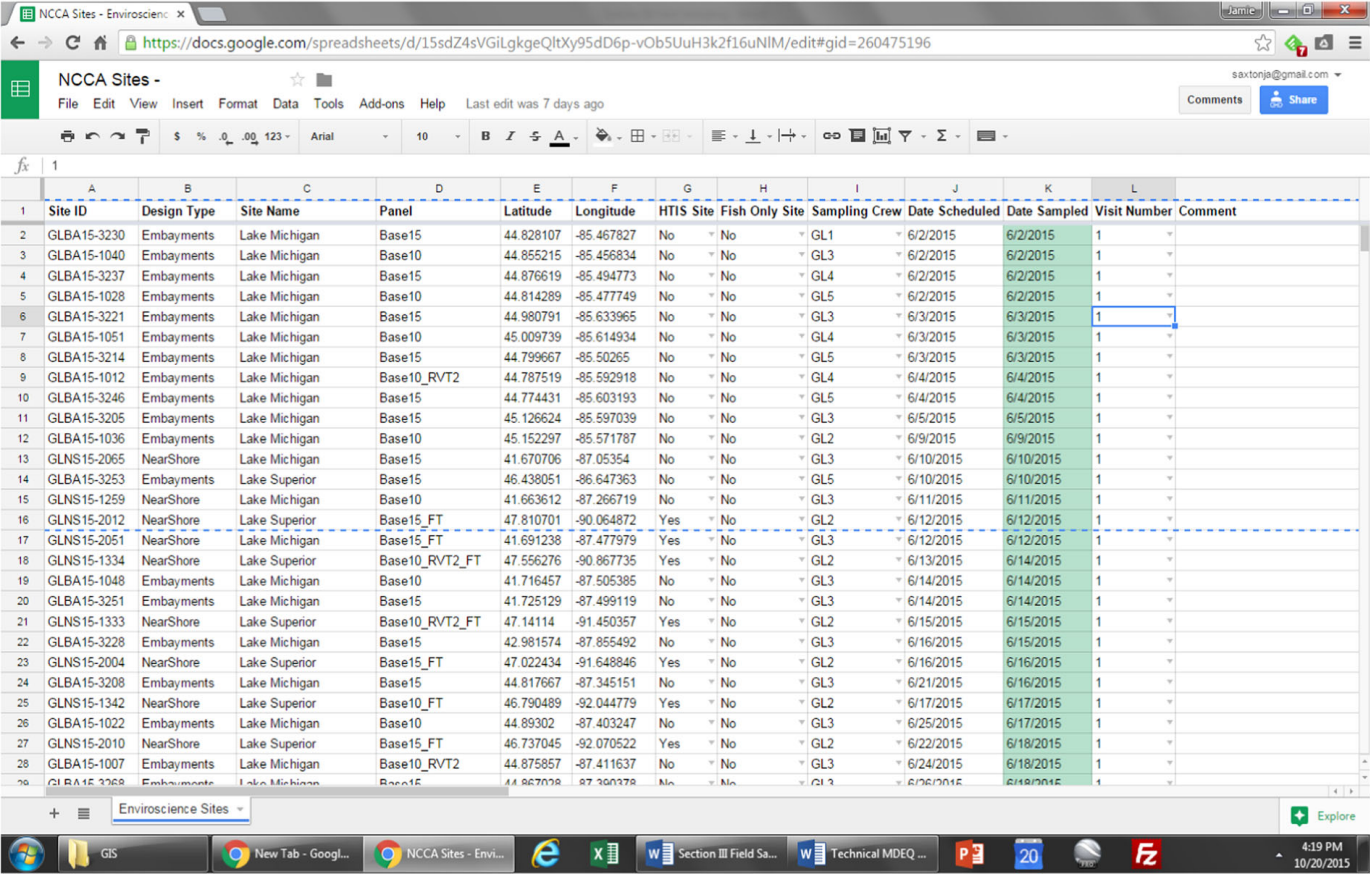
Screenshot of tracking sheet used to track sampling progress

Along with shipping logistics, the NARS logistics contractor and CFLC track and help manage all information concerning crew training and sampling progress during the field season. Standard operating procedures (SOPs) are followed to track the progress of the training season, sampling season, and the field crews. In compliance with these SOPs, the CFLC obtains data from status and tracking forms submitted by field crews, site evaluation spreadsheets, and other communications; compiles data into a spreadsheet to retain an independent list of sites sampled and samples collected; and uses the data to generate regularly updated summary charts that allow USEPA staff and other stakeholders to monitor survey progress (Fig. 3). Information in tracking summary tables typically includes but is not limited to the total number of sites sampled, number of sites sampled per crew, number of each type of sample collected, reasons for dropped sites, reasons for failed sample collection, and replacements of revisit sites (Figs. 4 and 5).

**Fig. 6:**
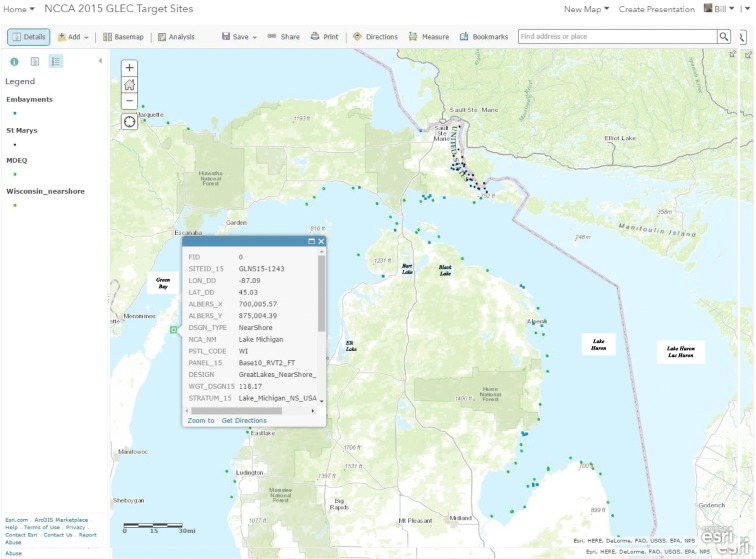
Screenshot of ArcGIS online sampling progress tracking map for the NCCA

For the NCCA, the logistics contractor developed a web-enabled tracking map (Fig. 6) using ESRI® ArcGIS Online to assist the CFLC, crew leaders, and project managers with field crew logistics. This tracking tool provided many benefits to the NCCA coastal assessment and is useful for similar surveys of lakes, rivers and streams, and wetlands. The online tracking map includes layers for each site category and is updated daily to indicate sampling status, and is generated from the aforementioned site tracking sheet updated daily by the CFLC. This adaptation made important improvements in survey efficiencies over prior surveys. Users can access and use the tracking map in an Internet browser from any location on any device. The online tracking map was used by the CFLC, field crew leaders, and project managers to obtain updates, in near-real time, on the status of sampling at assigned sites, which eliminated the possibility of accidently sampling a location twice. Additionally, the tool provides visual comparison of the location of sites, which field crew leaders may use in planning their sampling schedules. Once the tracking sheet is updated, the sampling locations are reclassified in a local ArcGIS map and the updated target site layers are then uploaded to the ArcGIS online map.

Tracking sampling progress allows the CFLC to detect protocol deviations, helps avoid missed sites and duplicated sampling events, and helps ensure that all sites are sampled within the sampling index period or allowable extension period. As crews complete the sampling season, they submit the USEPA-generated site evaluation spreadsheet to the CFLC. The CFLC reviews and compiles the information into a single spreadsheet, cross-checks the data to make sure evaluation questions were answered completely and appropriately, and submits the compiled data to USEPA. After completion of the field season, the CFLC cross-checks all site and sample details to ensure that the IM Database is accurate and that all samples are received by the laboratories. The CFLC also interacts with the laboratories to gather missing information pertaining to the samples (e.g., salinity values, filter volumes).

## Adaptive management for future national assessment logistics

The lessons learned and adaptive management practices applied by logistics contractors for USEPA’s NWCA and other national assessments have established the importance of learning and adaptation during the planning and execution of assessment survey seasons. Methods and manuals can be improved and updated as protocols are tested and reviewed in survey planning exercises. Field demonstration and proof-of concept trials serve to not only test protocols but also develop capable trainers and prepare field crews for the actual survey season. Communication tools and processes can be constantly improved by fine tuning procedures and adapting best in class technologies. Experience has proven, however, that future assessments will continue to be challenged by unanticipated events or environmental circumstances, both natural and social, which require adaptive management to maintain survey integrity. The logistical challenges encountered in previous surveys should guide adaptations of new tools and methods to control logistical constraints in future surveys.
